# Optimization of extraction of antioxidant polysaccharide from *Pleurotus ostreatus* (Jacq.) P. Kumm and its cytotoxic activity against murine lymphoid cancer cell line

**DOI:** 10.1371/journal.pone.0209371

**Published:** 2019-01-03

**Authors:** Md. Moyen Uddin Pk, Mohammad Sayful Islam, Rumana Pervin, Subhajit Dutta, Rabiul Islam Talukder, Matiar Rahman

**Affiliations:** 1 Institute of Biological Science, University of Rajshahi, Rajshahi, Bangladesh; 2 Department of Biochemistry & Molecular Biology, University of Rajshahi, Rajshahi, Bangladesh; 3 School of Science, Independent University of Bangladesh, Dhaka, Bangladesh; 4 Department of Biochemistry, Primeasia University, Dhaka, Bangladesh; 5 Department of Pharmacy, Mawlana Bhashani Science and Technology University, Tangail, Bangladesh; 6 School of Science, Banaras Hindu University, Varanasi, India; 7 Department of Clinical Biochemistry, Popular Diagnostic Centre Ltd., Dhaka, Bangladesh; College of Agricultural Sciences, UNITED STATES

## Abstract

The purpose of this study was to optimize the extraction method for polysaccharide from the fruiting bodies of *Pleurotus ostreatus* (Jacq.) P. Kumm and to assess the antioxidant and cytotoxic potentials of polysaccharide. In this investigation, polysaccharides from *Pleurotus ostreatus* (Jacq.) P. Kumm were extricated by utilizing the hot water. One-single factor and response surface methodology was established to optimize the extraction conditions for polysaccharide from *Pleurotus ostreatus* (Jacq.) P. Kumm. Examination of antioxidant activity of *Pleurotus ostreatus* polysaccharide (POP) was directed by utilizing 2, 2-diphenyl-1-picrylhydrazyl (DPPH) and 2, 2-azino-bis-3-ethyl-benzothiazoline-6-sulfonic acid (ABTS) techniques. Cytotoxicity of POP was evaluated using an MTT assay. The experimental data were fitted to a quadratic equation utilizing multiple regression investigations, and the ideal conditions were as per the following: water/crude material proportion, 26.04 mL/g; an extraction time of 62.08 minutes; and an extraction temperature 70.5°C. Under such conditions, the polysaccharide yield was 5.32 ± 0.12% with the anticipated yield. POP showed good scavenging activity against DPPH radical (p<0.001, EC_50_ = 1036.38 μg/mL, R^2^ = 0.8313) and ABTS radicals (p<0.001, EC_50_ = 824.37 μg/mL, R^2^ = 0.8223), with a dose (p<0.001)-and-time (p<0.001) dependent cytotoxic potential on Ehrlich ascites carcinoma cell line in vitro. This demonstrated that polysaccharides (POP) had certain cancer prevention agent exercises. In this manner, these examinations give reference to additionally research and reasonable improvement of *Pleurotus ostreatus* (Jacq.) P. Kumm polysaccharide and POP may prove a useful therapeutic agent, due to its robust antioxidant and cytotoxic activity.

## Introduction

*P*. *ostreatus*, the oyster mushroom, is a consumable mushroom, generally growing all over the world. Currently, it serves as a rich source of nourishment[1]. Oyster mushrooms are a rich source of vitamins, amino acids, and minerals, as well as antioxidants[2]. In addition to their role in nutrition, they possess medicinal properties. Recent research has elucidated the essential role of Oyster mushroom՛s extracts in lowering blood cholesterol[3], scavenging free radical[4] and inhibiting enzyme activity[5]. In recent years, there has been an increasing interest in the extraction and characterizations of polysaccharides from edible mushrooms. No previous study has investigated on polysaccharides extraction and study of biological activities from *Pleurotus ostreatus*. Polysaccharides, are a kind of macromolecule sugar polymer, found in a large number of plants and fungus [6–8]. Specialists have found that polysaccharides extricated from various plants possess compelling properties [9–11]. These properties include antitumor, antiviral activity, immunomodulation, antilipidemic impact and oxidation protection [12–14]. In order to harbor the full potential of the polysaccharides, the polysaccharide must be proficiently extracted from Oyster mushrooms. The proficient extraction will enable the advancement and use of this fungus on a wider scale. In recent times, there have been a few investigations centered on the extraction of polysaccharide from Oyster mushrooms. Extracting polysaccharide with hot water is the most widely recognized method utilized in the agricultural industry, because of its easy accessibility and safety [15]. By and large, extraction proficiency of polysaccharide is influenced by several factors: extraction time, extraction temperature and water to material proportion. Their effects on the polysaccharide yield may be autonomous. The response surface methodology (RSM) determines the optimum conditions for extracting polysaccharides. The RSM procedure enables the interplay of several factors relating to polysaccharide yield to be investigated simultaneously. RSM creates a polynomial equation and an inclined model by multiple regression fitting of response surface examination. The RSM enables the extent of the polytomy factors in influencing polysaccharide yield and to be assessed, and their optimum level to maximize yield[16,17]. RSM has been effectively used to optimize different biochemical and biotechnological processes[18]. Box-Behnken Design, a kind of RSM, was used to find optimum extraction parameters of polysaccharides extraction from *Pleurotus ostreatus*. The objective of this investigation was to optimize the conditions for polysaccharide extraction from *Pleurotus ostreatus* (Jacq.) P. Kumm and to find the ideal extraction condition (water to crude material ratio, extraction time and extraction temperature) utilizing response surface methodology. The in vitro antioxidant activities of POP were assessed on the base of 1,1-diphenyl-2-picrylhydrazyl (DPPH) radical scavenging activity and 2,2-azino-bis-3-ethyl-benzothiazoline-6-sulfonic acid (ABTS) radical scavenging activity. Additionally, the 3-(4, 5-dimethylthiazol-2-yl)-2, 5-diphenyl tetrazolium bromide (MTT) assay was used to measure in vitro role of cytotoxicity in Murine Lymphoid cancer cell line (EAC cell line).

## Materials and methods

### Materials and reagents

Fresh fruiting bodies of *Pleurotus ostreatus* (Jacq.) P. Kumm were collected and authenticated from the Mushroom Development Institute, which belongs to the Department of Agriculture Extension, Ministry of Agriculture, Savar, and Dhaka-1340 in Bangladesh. Monosaccharide (D-glucose), vitamin C, 1,1-diphenyl-2-picrylhydrazyl (DPPH), 2,2-azino-bis-3-ethyl-benzothiazoline-6-sulfonic acid (ABTS), thiazolyl blue tetrazolium bromide (MTT), trichloro acetic acid (TCA), alfa-Amylase, bovine serum albumin (BSA), acetonitrile, 1-phenyl-3-methyl-5-pyrazolone (PMP), RPMI-1640 were purchased from Sigma Co., St. Louis, MO, USA, while sephadex-G-100 and DEAE cellulose were acquired from Pharmacia, Co., Sweden. Murine Lymphoid cancer cell line or Ehrlich ascites carcinoma (EAC) cells were obtained with the courtesy of Indian Institute of Chemical Biology, Kolkata, India. Dimethyl sulfoxide, chloroform, butanol, sodium chloride, phenol, sulfuric acid, uronic acid, glucuronic acid were purchased from local agents (Diatech, Alfa Scientific Co. and Bio-trade BD).

### Extraction of *Pleurotus ostreatus* (Jacq.) P. Kumm polysaccharide (POP)

POP was extracted by hot distilled water based on the intended conditions. After centrifugation (3000 rpm for 20 min), 1/3 of the original volume of the main supernatant was achieved by hot air concentration at 56°C. The starch in the concentrated solution was removed by alfa-amylase at 60°C. An adequate volume of ethanol was added to the concentrated extraction solution and mixed well to precipitate the POP at 4°C for 12 h. Then, the precipitates were gathered by centrifugation (3000 rpm for 20 min) and washed consecutively with acetone, ether and ethanol. The POP extract was liquefied with distilled water and protein was removed with Sevag reagent (chloroform: normal butanol, 4:1, v/v). The protein fractions from POP extract were removed by dialysis (MWCO 1400 Da, Union Carbide) and finally POP was collected by freeze drying. The POP yield was calculated using the following formula:

$$Y(\%)=\frac{Wpops}{Wsample}\times 100$$

Where, Wpops and Wsample are the weights of POP and *Pleurotus ostreatus* (Jacq.) P. Kumm powder used respectively.

### Single-factor experiment design

In this experiment, the following three variables were investigated: water to sample ratio (mL/g), extraction time (minutes), and extraction temperature (^o^C). Their variable effects on the POP extraction were assessed. Each sample was extracted according to the above mentioned procedure for polysaccharide extraction. The effects of three different conditions on POP yield were compared by one-way analysis of variance (ANOVA) using SPSS 21.0 (SPSS Inc., Chicago, IL).

### Design of Box-Behnken

The Box-Behnken design (BBD) was used to obtain the experiment design, analysis of results and regression models. Through the single factors experiment, the appropriate ranges of water to raw material powder (X_1_), extraction time (X_2_) and extraction temperature (X_3_) were determined and then the BBD of POP yield was carried out. The entire design was made up of 17 experimental runs, which were carried out in a random sequence. The three variables at the three levels were coded as -1, 0 and +1. Here, a second-order—polynomial formula was used to optimize the extraction conditions of the POP yield (the response) as follows [17]:

$$Y=A_{o}+\sum_{i=1}^{3}A_{i}X_{i}+\sum_{i=1}^{3}A_{ii}{X^{2}}_{i}+\sum_{i=1}^{2}\sum_{j=i+1}^{3}A_{ij}X_{i}X_{j}$$

Where, Y is the dependent variable and X_i_ X_j_ are the independent variables and A_o_, A_i_, A_ii_, and A_ij_ are the regression coefficients of the independent variables that were estimated by the model for intercept, linear, quadratic, and interaction terms, respectively. Design Expert 8.0.5.0 was utilized for statistical investigation of fluctuation for every response and anticipating the ideal conditions for the extraction of POP [19,20].

### Purification of polysaccharide from Pleurotus ostreatus (POP)

The dried POP was also deproteinated using the Sevage method[21] and 1.0 g of POP was dissolved in 10 mL dH_2_0 and applied to a DEAE-52 cellulose column (3.5 cm × 20 cm), and eluted with deionized water, followed by different gradient of NaCl solutions (0 to 0.5 M) at a flow rate of 0.8 mL/min. The eluates were collected in 5 mL fractions automatically and polysaccharide content was measured using phenol-sulfuric acid method[22]. The main identical fractions containing polysaccharide were collected and concentrated and further fractionated by using gel filtration chromatography on Sephadex G-100 column (110 cm × 1.6 cm) at 0.1 mL/min and eluted with distilled water. 4 mL fractions were collected per tube and polysaccharide content of each tube was measured using phenol-sulfuric acid method[22]. The major fractions were combined and lyophilized to yield POP, which was used for further studies.

### Analytical method validation

The polysaccharide content in POP extract was analyzed by phenol-sulfuric acid method using glucose as the standard. The regression equation was Y = 0.0231x – 0.046 and the correlation coefficient was 0.996. A linear relationship between the polysaccharide content and absorbance was detected within the range of 0–80 μg/mL, identified at 490 nm. The precision was appraised by analyzing the reproducibility and intermediate precision variations at three different concentrations with five replicates. The accuracy was evaluated with the pointed rescue test.

### Chemical composition analysis

The phenol-sulfuric acid method was used to determine the total polysaccharide content of POP extract, using D-glucose as the standard[22]. Protein content of POP extract was determined by Lowery method, using BSA as the standard[23]. The *m*-hydroxybiphenyl assay was used to measure uronic acid content of POP extract, using glucuronic acid as reference material [24]. The moisture content of POP was measured, referring to the method outlined in Kong et al (22). The pH values of the POP at the 2 mg/mL of POP were determined using a pH meter and the relative viscosity of POP (10 mg/mL) was determined using viscometer (Thermo Scientific HAAKE, 388–0100) at 25°C[25].

### Monosaccharide composition

The monosaccharide composition of the POP was determined using HPLC by methods determined by Chai, Y *et al*[26] with minor modifications. POP samples (2 mg) were hydrolyzed with trifluoroacetic acid (0.5 mL of TFA, 2 mol/L) in a wrapped flask occupied with nitrogen at 120°C and after 2 h of hydrolysis, the excess TFA from the flask was removed by evaporation with ethanol at 45°C. Monosaccharide standards and POP hydrolysate were then added to 0.5 mL of PMP (1-phenyl-3-methyl-5-pyrazolone, 0.5 mol/L in methanol) and 0.5 mL NaOH (0.3 mol/L) for derivatization at 70°C for 30 min. After centrifugation (10,000 rpm, for 5 min), the supernatant was assorted with 0.05 mL HCl (0.3 mol/L), and the mixture was mined with chloroform to eliminate surplus PMP. The aqueous layer was sieved using a 0.22 μm membrane and used for monosaccharide composition analysis of POP by HPLC system, which was furnished with a UV detector (245 nm) and an Amethyst C18 column (4.6 mm × 250 mm, 5 μm, Sepax, USA). The mobile phase of HPLC system was a mixture of PBS (Phosphate buffered saline, 0.1 mol/L, pH 7) and acetonitrile (80:20, v/v) with a flow rate of 1 mL/min. The column was reserved at 25°C, and the injection volume was 10 μL.

### UV-Vis and FT-IR analysis

The purified POP was re-dissolved in water to achieve a concentration of 0.25 mg/mL. The UV-Vis spectra of the solution were recorded at 190–600 nm, using spectrophotometer (Double beam UV-visible light spectrophotometer, China) and the functional groups of POP were acknowledged using FT-IR spectrophotometer, within 4000–500 cm^-1^in KBr pellets.

### DPPH radical scavenging activity

DPPH radical scavenging activity of POP was determined according to a modified method of Sharma *et al* 2016 [27]. Vitamin C was used as the positive control. Briefly, 50 μL of each POP samples ranging in concentrations between 0.2 to 1.2 mg/mL were prepared by dissolving POP solid in distilled water and then adding a 5 mL (0.004% w/v in ethanol) solution of DPPH. The mixture was vortexed and shielded with Aluminum foil and incubated for 30 min in room temperature in the dark environment. The blank control was 80% ethanol and measurements were taken in triplicate. After 30 minutes of incubation, discoloration of reaction was measured using spectrophotometer at 517 nm. DPPH scavenging effect was determined using the following equation;

$$DPPH\;scavenging\;effect\;(\%)=\frac{[A_{o}-(A_{1}-A_{2})]\times 100}{A_{o}}$$

Where, Ao is the absorbance of the control; A_1_ is the absorbance of the POP sample; A_2_ is the absorbance of the POP sample under similar condition to A_1_ excepting ethanol instead of the DPPH. The effective concentration for 50% of free radical scavenging activity of positive control (Vitamin C) and POP samples were determined using standard curve (r^2^ = 0.9994).

### ABST radical scavenging assay

The ABST radical scavenging of POP was carried out using the method of P. Li *et al* 2011[28]. Vitamin C was used as the positive control. The ABTS radical cation was produced via the reaction between ABTS solution (5 mL, 7 mM) and K_2_S_2_O_8_ aqueous solution (1 mL, 15 mM) following 12 h in the dark. The ABTS radical cation solution was then diluted with deionized water to yield an absorbance of 0.70 at 734 nm. The ABTS radical cation answer (3 mL) was added to 0.75 mL POP solution (dissolved in distilled water) at varying concentrations (0.1, 0.2, 0.4, 0.6, 0.8, 1.0 and 1.2 mg/mL). After 15 minutes of reaction, the absorbance was measured at 734 nm. The scavenging endeavor of POP in opposition to ABTS radical was evaluated with the aid of the accompanied equation:

$$ABST\;radical\;scavenging\;activity\;(\%)=\frac{[A_{0}-(A_{1}-A_{2})]\times 100}{A_{0}}$$

Where, A_o_ is the absorbance of the control; A_1_ is the absorbance of the POP solutions; and A_2_ is the absorbance of all the reaction reagents (Except POP solutions).

### Determination of cytotoxic activity of POP using MTT assay

Murine Lymphoid Cancer Cell Line or EAC (Ehrlich ascites carcinoma), which are murine lymphoid cancer cell lines were cultivated in RPMI-1640 medium (10% foetal calf serum, and 1% (v/v) penicillin-streptomycin) in a humidified atmosphere of 5% CO_2_ at 37°C. EAC cells were planted (5 ×10^3^ cells/well in 200 μL RPMI 1640 media) in 96-wells, with various concentrations of purified POP (0.001–1000μg/mL) added and incubated for 24, 48, and 72 hrs at 37°C in 5% CO_2_ atmosphere. After the end of incubation, the medium was removed and wells were washed with 10 mM PBS and MTT (3-(4, 5-dimethylthiazol-2-yl)-2, 5-diphenyl tetrazolium bromide) (20 μL, 5 mg/mL MTT in PBS) was added and hatched for 4 h at 37°C. 100 μL of acidic isopropanol was added into each well and incubation continued for 3 h more. The absorbance was measured at 570 nm and the proliferation rate (PR) was determined using the following formulae:

$$PR=\frac{Tabs}{Cabs}\times \;100$$

Where, Tabs and Cabs are the absorbance of test and control. Cytotoxicity of POP on EAC cells was determined as cells growth inhibition rate (IR):

IR=100−PR

### Statistical analysis

GraphPad Prism 6 (Pad Software, Inc., USA) was used to analyze data, and results are expressed as mean±SD. Error bars were expressed as 95% CI. All experiments were performed at least three times. *P*<0.05 was considered to be statistically significant, and *P*<0.01 was regarded as highly statistically significant. Design Expert Software (Version 9.0.4.1, State-Ease Inc., USA) was used to design of Box-Behnken and single-factor experiment.

## Results and discussion

### Impact of water to raw material ratio on the POP yield

**Fig 1:**
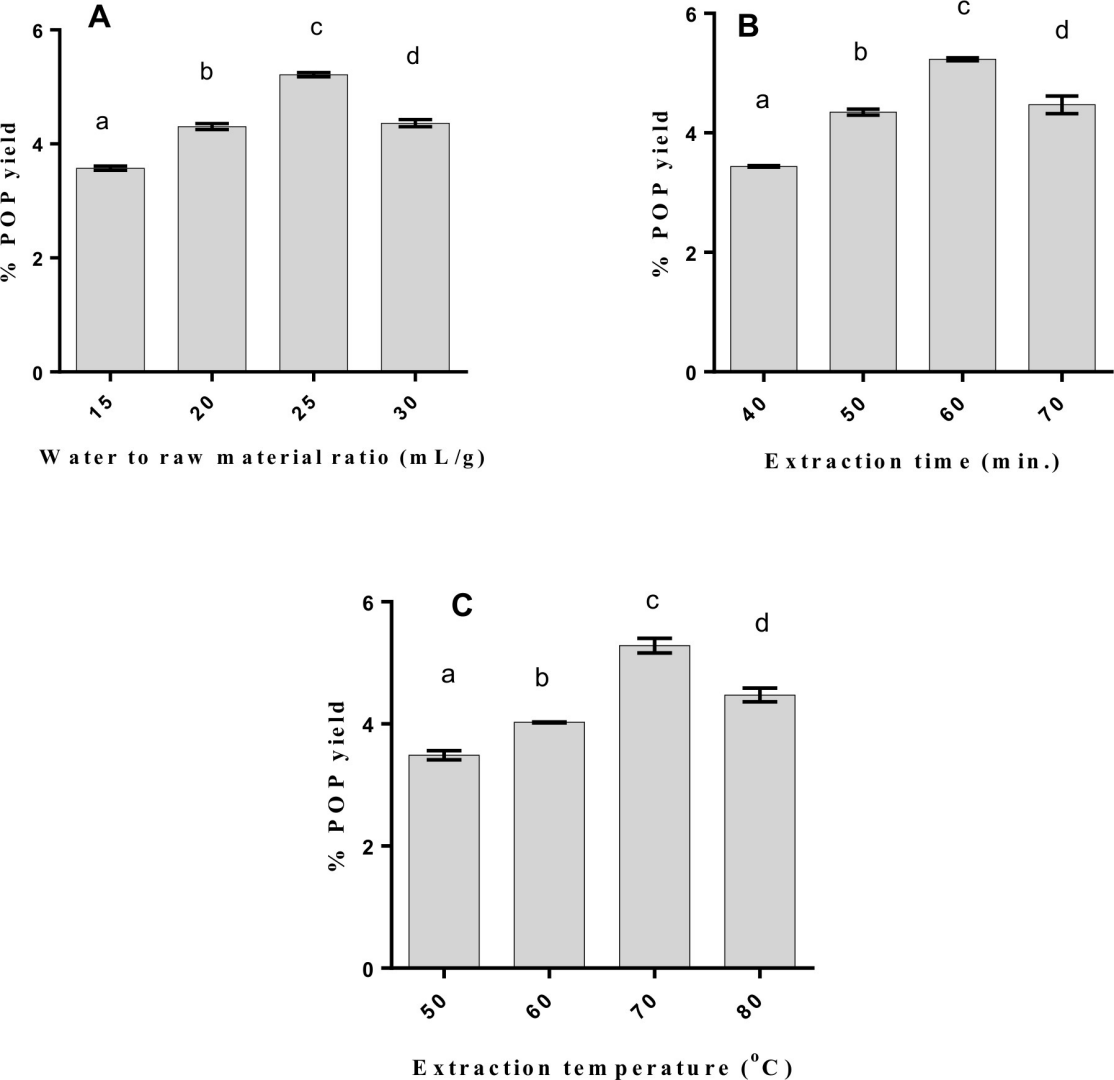
**Effect of water to raw material ratio (A), extraction time (B), and extraction temperature (C) on POP yield.** Bar charts with different lowercase letters significantly differ (p<0.05). POP, *Pleurotus ostreatus* polysaccharide. The % yield of POP increased from 3.57% to 4.36% and increased to 5.21% with an increasing water to raw materials ratio, from 15 mL/g to 25 mL/g. However, when the water to crude material proportion increased to 30 mL/g there was a reduction in the extraction yield of POP. As the water to crude material proportion increased, the disintegration of polysaccharide from the materials essentially increased, and the extraction yield of POP increased. In this way, the water to crude material proportion of 25 mL/g was selected for the BBD analysis.

The polysaccharide was extracted at different water to raw materials ratios, with a fixed extraction time and extraction temperature at 60°C and 70 min respectively. This allowed the influence of water to raw materials ratio on the percent yield of POP to be evaluated (Fig 1A).

### Impact of extraction time on the POP yield

Extraction time is a vital parameter, influencing the extraction yield of polysaccharide. To research the impact of extraction time on the extraction yield of POP (Fig 1B), the polysaccharide was removed at various extraction time (40 50, 60, and 70 min), and the water to crude material proportion and extraction temperature were set as 25 mL/g and 70°C, separately. The extraction yield of POP essentially increased from 3.44% to 5.23% when the extraction time increased from 40 to 60 min (P < 0.05). However, when this extraction time was exceeded, the extraction yield of POP only decreased marginally. Consequently, the extraction time of 60 min was selected for the BBD analysis.

#### Impact of extraction temperature on the POP yield

To assess the impact of extraction temperature on the yield of POP (Fig 1C), the water to crude material proportion and extraction time were kept at 25 mL/g and 60 min, individually. The extraction yield of POP increased from 3.49% to 5.28% when the temperature increased from 50 to 80°C, and the POP yield at 70°C was essentially higher than those of 50, 60 and 80°C (P < 0.05). In any case, the extraction yield of POP at 80°C was marginally lower than that of 70°C (P > 0.05). For this reason, the extraction temperature of 70°C was chosen selected for the BBD analysis.

### Optimization of polysaccharide extraction from *Pleurotus ostreatus*

Based on the single-factor tests, 17 runs were performed to streamline the extraction states of POP. The POP yields (%) of the 17 runs designed to assess the 3 autonomous factors including water to crude material proportion (X1), extraction time (X2) and extraction temperature (X3) are exhibited in Table 1. The POP yields range from 3.44% to 5.28%. The percentage of POP yield (% Y) was described by the 2^nd^ order polynomial Eq (1) as follow:

$$\begin{array}{l} Y\;\left(\%\right)=-58.88+1.52725\;X_{1}+0.677375\;X_{2}+0.649125\;X_{3}+0.000775\;X_{1}X_{2}‑0.001475\\ X_{1}X_{3}+0.000262\;X_{2}X_{3}‑0.028825\;X_{1}{}^{2}‑0.005881\;X_{2}{}^{2}‑0.004181\;X_{3}{}^{2} \end{array}$$ (1)

Where, Y is the POP yield; X1, X2 and X3 are the factors of water to crude material proportion, extraction time and extraction temperature, individually.

**Table 1:** Box-Behnken experiment design and the results of the POP yield.

Run	Water to raw material ratio (X_1_) (mL/g)	Extraction time (X_2_) (min)	Extraction temperature (X_3_) (^o^C)	POP yield (%)	
				Actual value	Predicted value
**1**	25	60	70	5.41	5.39
**2**	30	50	70	4.105	4.09
**3**	20	70	70	3.98	3.99
**4**	30	70	70	4.365	4.36
**5**	25	60	70	5.4	5.39
**6**	25	60	70	5.395	5.39
**7**	20	50	70	3.875	3.88
**8**	30	60	80	4.735	4.75
**9**	25	60	70	5.425	5.39
**10**	25	70	80	4.94	4.93
**11**	25	50	80	4.695	4.69
**12**	30	60	60	4.045	4.04
**13**	25	50	60	3.88	3.89
**14**	25	60	70	5.32	5.39
**15**	20	60	60	3.62	3.60
**16**	25	70	60	4.02	4.02
**17**	20	60	80	4.605	4.60

POP, *Pleurotus ostreatus* polysaccharide.

The aftereffect of ANOVA investigation is displayed in Table 2. The importance of coefficients can be verified by their respective *P*-values, and a smaller *P*-value signifies a more significant comparing coefficient. In Table 2, the model *P-*value was less than 0.0001, which demonstrates that the relapse display for POP yield was highly significant. Furthermore, the *P*-estimations of the straight coefficients (X_1_, X_2_ and X_3_), and the quadratic term coefficients (X_1_^2^, X_2_^2^ and X_3_^2^) were less than 0.01, which shows the significant effects of these three variables on the extraction yield of POP. The *P*-estimation of the communication term coefficient (X_1_X_3_) was observed to be lower than 0.05, showing the critical impact of this coefficient on the extraction yield of POP. The other term coefficients (X_1_X_2_, X_2_X_3_) were not significant (P > 0.05). The ANOVA shows that water to crude material proportion (X_1_), extraction time (X_2_) and extraction temperature (X_3_) were significant single factors influencing the extraction yield of POP.

**Table 2:** ANOVA analysis for the quadratic model of POP extraction.

Source	Sum of Squares	df	Mean Square	F-value	p-value
**Model**	6.59	9	0.7319	674.13	< 0.0001**
**A-X1**	0.1711	1	0.1711	157.60	< 0.0001**
**B-X2**	0.0703	1	0.0703	64.76	< 0.0001**
**C-X3**	1.45	1	1.45	1338.76	< 0.0001**
**AB**	0.0060	1	0.0060	5.53	0.0509
**AC**	0.0218	1	0.0218	20.04	0.0029**
**BC**	0.0028	1	0.0028	2.54	0.1551
**A**^**2**^	2.19	1	2.19	2013.91	< 0.0001**
**B**^**2**^	1.46	1	1.46	1341.41	< 0.0001**
**C**^**2**^	0.7361	1	0.7361	678.01	< 0.0001**
**Residual**	0.0076	7	0.0011		
**Lack of Fit**	0.0010	3	0.0003	0.1905	0.8978
**Pure Error**	0.0066	4	0.0017		
**Cor Total**	6.59	16			
**R**^**2**^	0.9988				
**Adjusted R**^**2**^	0.9974				
**Predicted R**^**2**^	0.9961				
**Adeq Precision**	70.6326				
**C.V. %**	0.7199				

**p<0.001 was measured to be highly significant.

The assurance coefficient (R^2^) of the model was 0.9988, showing that 0.12% of aggregate varieties couldn't be clarified by the model. However the balanced assurance (adj-R2) esteem (0.9974) showed that a large portion of the POP yield variety could be anticipated by the relapse demonstrate. The coefficient of variety (C.V.) was at low esteem (0.7199%), demonstrating the investigation information and predicated information were comparative. As per the exploratory information, the lack of fit *P*-value (0.8978) was more than 0.05, proposing that the lack of fit was unimportant in identifying the pure error. The Adeq Precision (70.6326) in the present model was a sufficient flag, which demonstrated that the model could be utilized to explore the plan space.

### Optimization of extraction conditions

**Fig 2:**
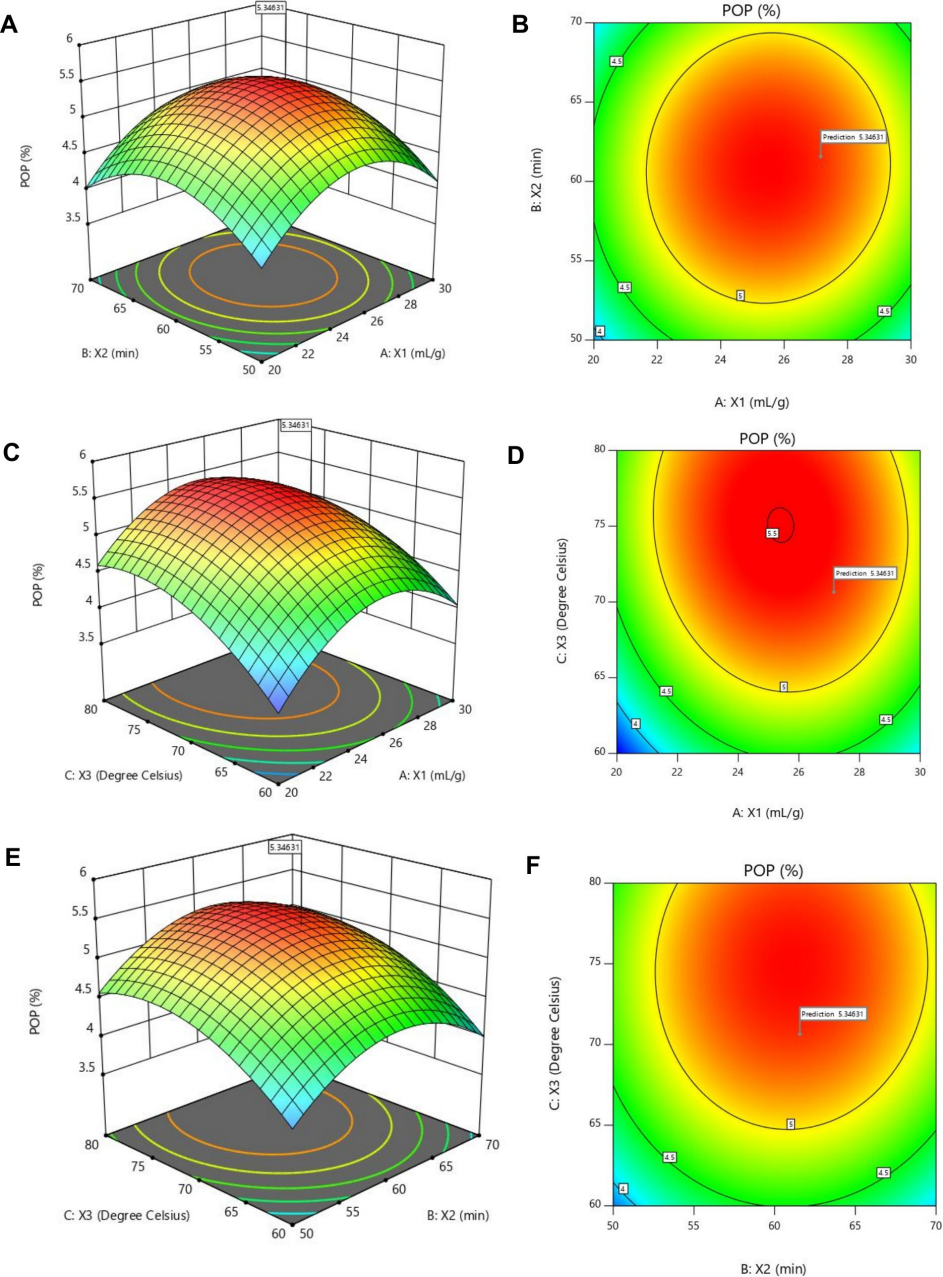
Response surface and contour plots for % yield of POP from *Pleurotus ostreatus* (Jacq.) P. Kumm. (A-B) Water to raw materials and extraction time (C-D) Water to raw materials and extraction temperature and (E-F) Extraction time and extraction temperature.

The impacts of three variables on POP yield (%Y) were analyzed with three-dimensional response surface plot and contour plot, as shown in Fig 2. In this experiment, two variables were presented by response surface plot and contour plot whereas a variable was fixed at the 0 level. For water to crude material proportion and extraction time (Fig 2B), the contour plot is circular, which demonstrates that the shared connection between water to crude material proportion and extraction time is not significant (P > 0.05). The comparative pattern (Fig 2F) was found for extraction time and extraction temperature (P > 0.05). The elliptical contour plot (Fig 2D) showed the common cooperation between water to crude material proportion and extraction temperature was noteworthy (P < 0.05). These outcomes were in concurrence with the qualities in Table 2.

### Diagnostics accuracy

**Fig 3:**
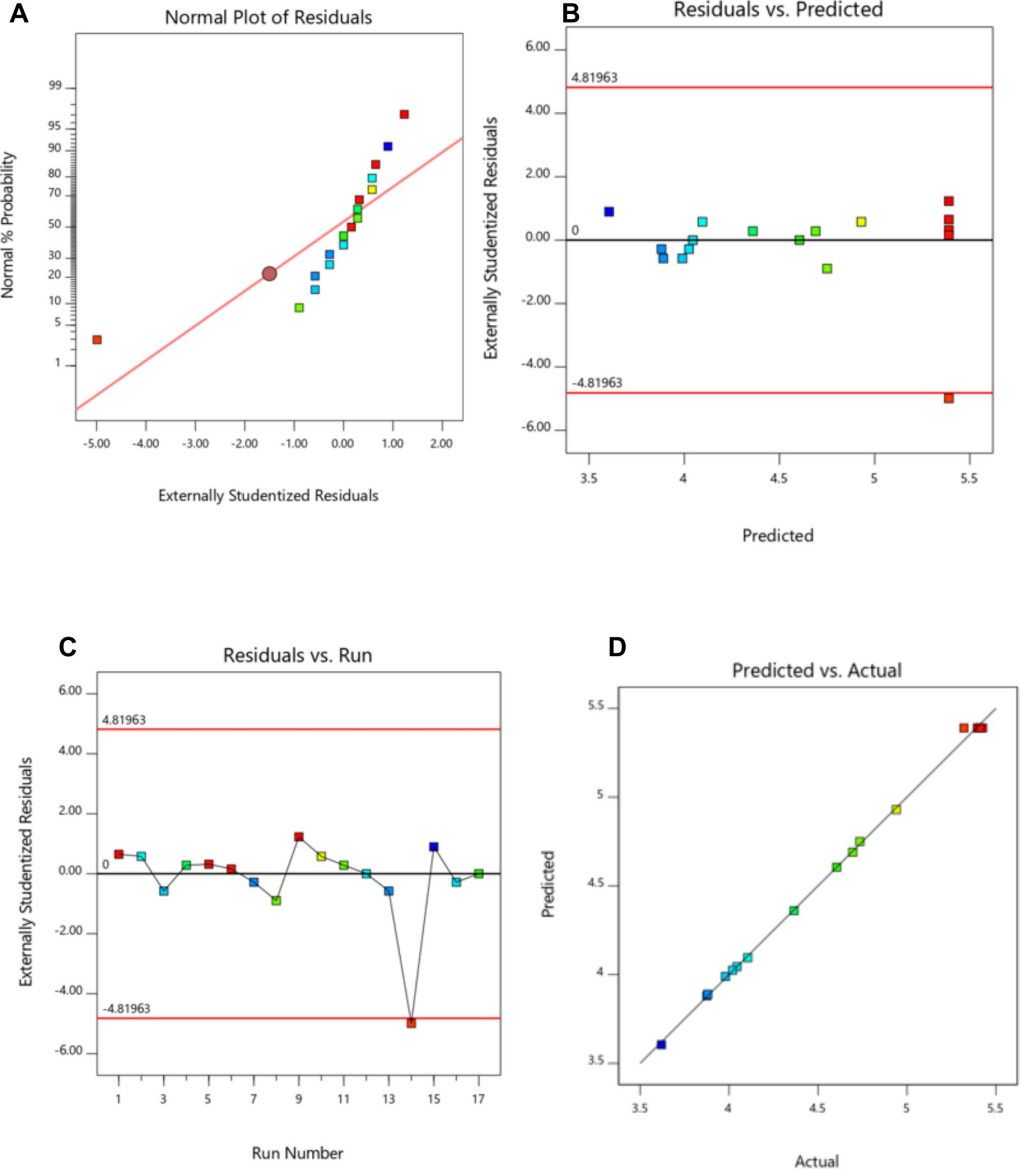
Diagnostic plots for the Box-Behnken model adequacy. (A) Normal plot of residuals, (B) plot of internally studentized residuals vs. predicted response, (C) Residual vs. run and (D). Predicted vs. actual.

Diagnostics accuracy is crucial when determining the appropriateness of the model for the genuine framework. Four diagnostics graphs for model accuracy are displayed in Fig 3. Fig 3D demonstrates the anticipated and the genuine test esteems. The spots of the anticipated and real esteems indicates ordinary circulation and are near the 45° line, confirming that the model has a decent adjustment. Fig 3A demonstrates a normal % probability plot of residuals for the ordinariness presumption; the lingering plot that moves towards a straight line, demonstrating that the typicality supposition was appropriate. The inside studentized residuals versus anticipated esteems are shown in Fig 3B. The plots of the inside studentized residuals scattered arbitrarily demonstrate that the first fluctuation was steady for all esteems. The inside studentized residuals versus trial run numbers are displayed in Fig 3C, and every one of the focuses are situated inside a restricted range. All information shows that the reaction surface model is connected to the POP extraction, and that the model is critical and exact.

### Validation of the model

The ideal extraction conditions for the extraction of POP comprised of a water/crude material proportion of 26.04 mL/g, an extraction time of 62.08 minutes, and an extraction temperature 70.5°C, which were determined by utilizing the second-order quadratic equation of the model by fathoming the regression equation and dissecting the response surface. The greatest anticipated estimation of POP yield was 5.34%. With a specific end goal to verify the anticipated estimation of POP yield and the applicability of the model, three parallel tests were performed under the previously mentioned ideal extraction conditions. The yield of POP was 5.32 ± 0.12% (n = 3), which drew nearer the anticipated estimation of 5.34%. The outcomes demonstrated that the RSM was fitting for enhancing the conditions for POP extraction, and the regression model was precise and relevant for extricating POP.

### UV and FT-IR analysis

**Fig 4:**
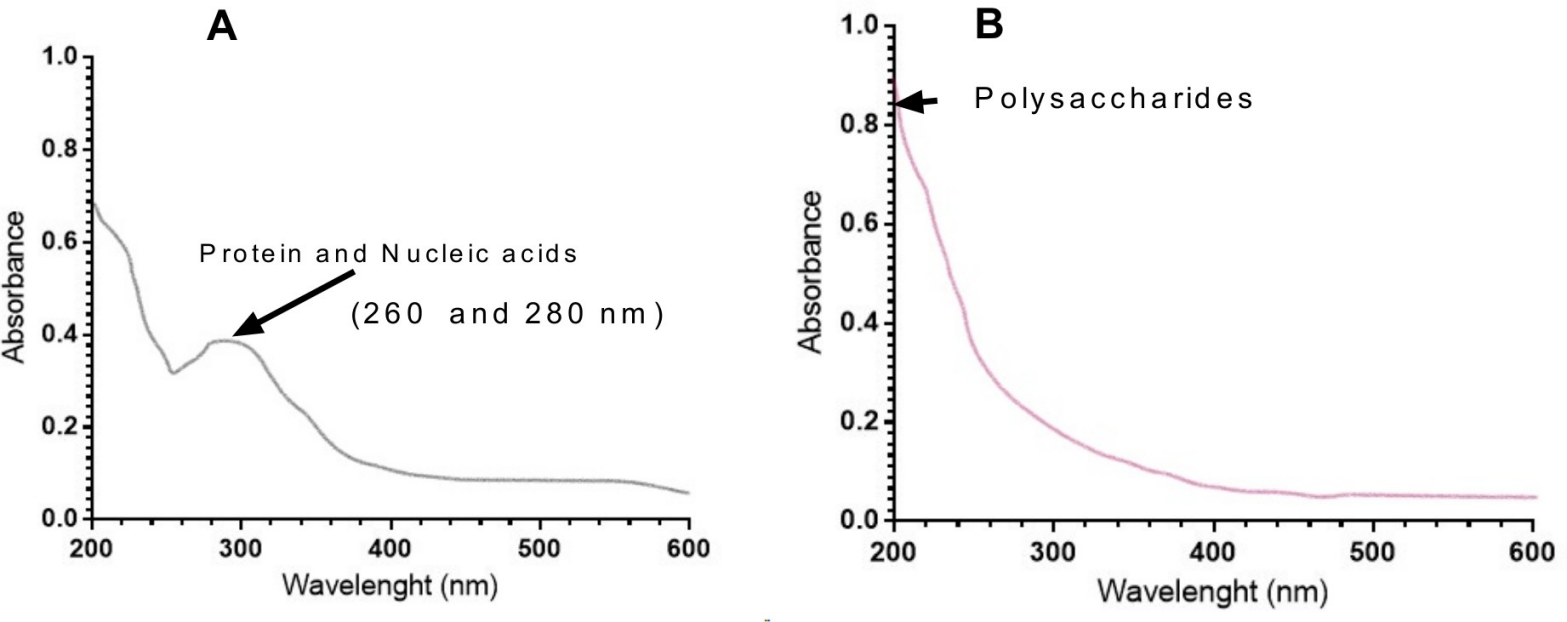
UV-spectrophotometric analysis of partially purified polysaccharides (POP). A) Before proteolysis and B) after removing of protein and nucleic acids. The peaks in the range of 950–1200 cm^-1^ indicate the presence of polysaccharides, indicating the presence of glycosidic bonds by 1,150–1,160 cm^-1^.

**Fig 5:**
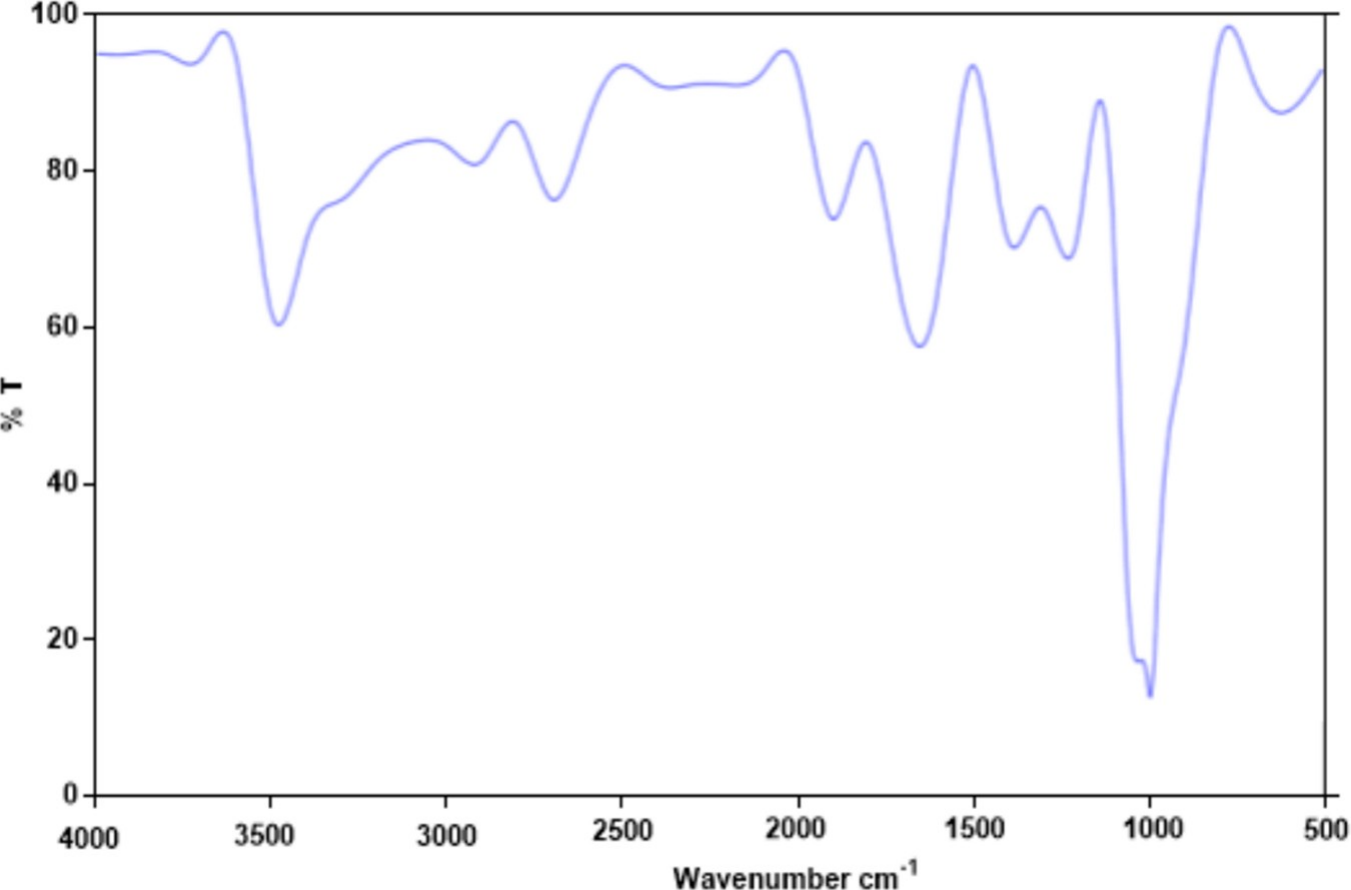
FT-IR spectral analysis of the polysaccharide (POP). The spectra indicate a noticeable C-H stretching and bending vibration absorption in the range of 2929–2939 cm^-1^, which are characteristic peaks of the polysaccharide[29]. In the marked region, the peak in the region of 1230 cm^-1^ is due to C-C stretching vibration and the peaks towards 1100 cm^-1^ suggesting that the peak was related to the starching vibration of C-O.

Fig 4A and 4B show the UV-visible absorption spectra of polysaccharide. Fig 4B exhibits that polysaccharide fraction have no absorption at 260 and 280 nm in the UV spectrum, signifying the absence of nucleic acid and protein. The IR spectra of POP are shown in Fig 5.

### Chromatographic analysis

**Fig 6:**
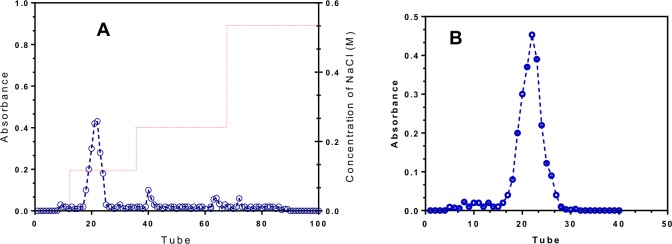
Chromatographic profile of POP. A) The elution profile of POP on DEAE-cellulose column and B) gel filtration elution curve of POP. POP, *Pleurotus ostreatus* polysaccharide.

A portion of POP (10g) was separated through a DEAE-Cellulose column and three portions were collected according to the chromatography profile (Fig 6A). A portion of POP was further purified on gel filtration chromatography and a polysaccharide (POP) was obtained (Fig 6B).

### Chemical and sugar composition

The monosaccharide composition analysis indicates that POP was composed of only glucose, confirming that it was a glucan. Table 3 shows the chemical and sugar composition of POP.

**Table 3:** Chemical and sugar composition of POP.

		POP
**Total sugar content (%)**		97.6±1.33
**Protein content (%)**		0.5±0.03
**Uronic acid content (%)**		nd^a^
**pH**		7.41±0.04
**Moisture content (%)**		14.11±0.27
**Relative viscosity**		1.13±0.02
**MW (kDa)**		154.6
**Monosaccharide** **composition (%)**	**Glc**	100.0
	**Ara**	nd^a^
	**Gal**	nd^a^
	**Man**	nd^a^
	**Rha**	nd^a^

Values are expressed as mean±standard deviation. nd^a^, Not Detected. POP, *Pleurotus ostreatus* polysaccharide. Glc, Glucose; Ara, Arabinose; Gal, Galactose; Man, Manose; Rha, Rhamnose

The low moisture (14.11%) and viscosity (1.13) contents of POP confirmed that it is suitable for large scale production. The gel filtration analysis results showed that POP was a single regular peak (Fig 6B), indicating that it was an identical polysaccharide. The molecular weight of POP was calculated as 154.6 × 10^3^ k.

### In vitro antioxidant activity of POP obtained at optimum temperature, time and water to raw material ratio

**Fig 7:**
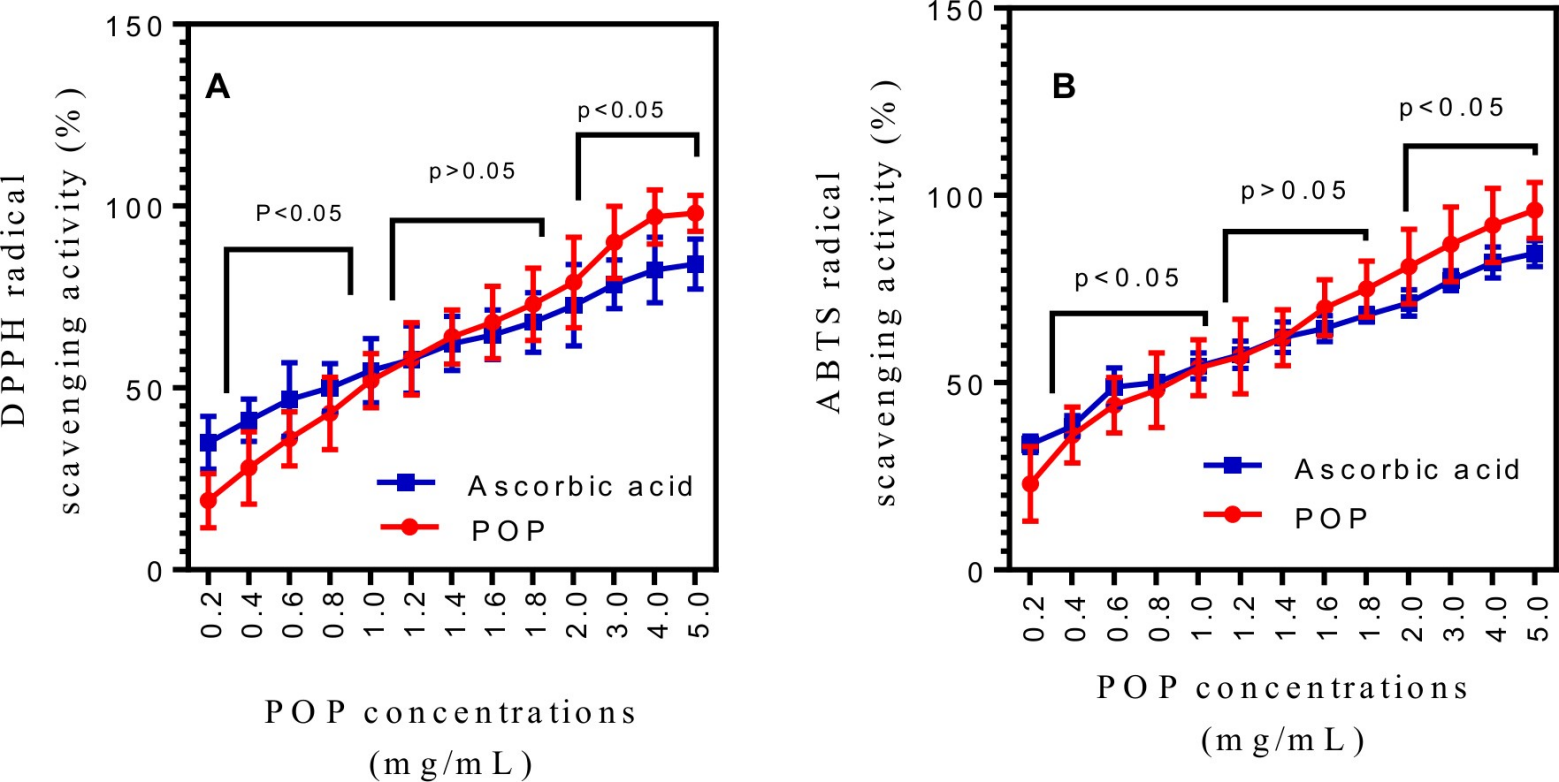
Antioxidant activities of the POP. (A) DPPH radical scavenging activity (p<0.001, EC_50_ = 1038.38 μg/mL, R^2^ = 0.8313) and (B) ABTS radical scavenging activity (p<0.001, EC_50_ = 824.37 μg/mL, R^2^ = 0.8223). POP, *Pleurotus ostreatus* polysaccharide.

The scavenging activity of POP on DPPH radical assay has been extensively used to determine the antioxidant capacity of POP due to its easy availability and the immovability of DPPH radicals [30]. DPPH is a steady radical that focuses on nitrogen and demonstrates a greatest consumption at 517 nm in methanol [31,32]. The scavenging ability of POP on DPPH radical and ABTS radical was shown in Fig 7A and 7B.

The results show the DPPH radical scavenging activities of POP increase gradually as the concentration of polysaccharides increases from 0.2 to 5.0 mg/mL. The POP demonstrates a concentration dependent free radical scavenging activity, with a significant activity found at higher concentrations. The outcomes demonstrate that the scavenging activity of DPPH in POP is low compared to that of vitamin C below 1.4 mg/mL (P<0.05) and in case of ABTS that concentration is 1.6 mg/mL (P<0.05). DPPH radicals scavenging by POP and vitamin C is equal at 1.2 mg/mL (72.23%) while at 1.4 mg/mL (81.02%) POP exhibit the same ABTS radicals scavenging activity, in comparison to vitamin C. Polysaccharides from plants and fungus have been perceived to be a promising source of antioxidant molecules. Numerous investigations have demonstrated that polysaccharides enhance the action of cell reinforcement proteins in the body, scavenge free radicals, and repress lipid oxidation [33].

### In vitro cytotoxic measure of POP using MTT assay

**Fig 8:**
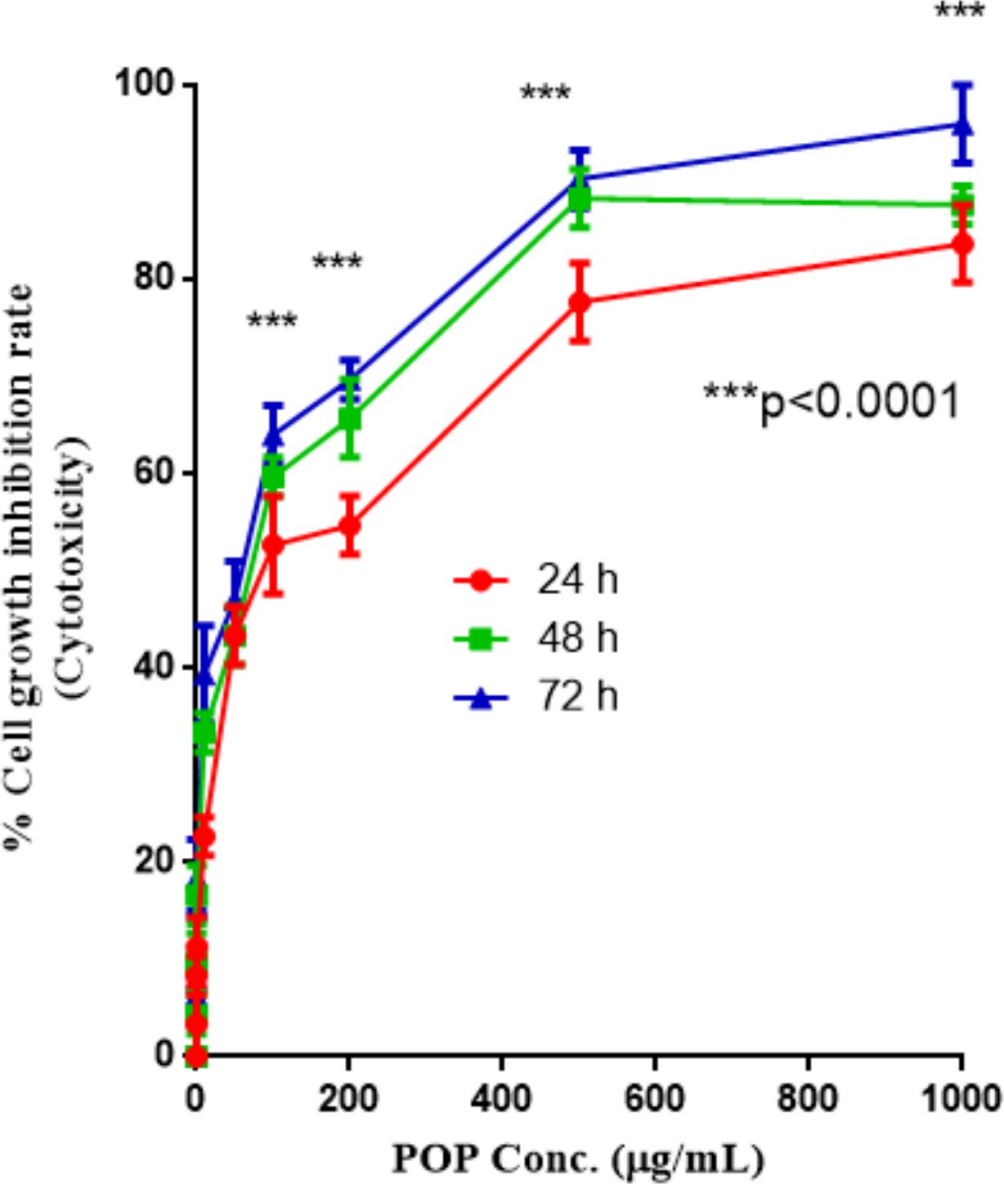
Cytotoxicity of purified POP on murine lymphoid cancer cell line (EAC cell line). POP, *Pleurotus ostreatus* polysaccharide.

The cytotoxic properties of purified POP was assessed by using the EAC cell line. The POP extract at 10^−3^ to 10^3^ μg/mL exhibit dose-and-time dependent inhibitory effects on the proliferation of EAC cells, with greater than a 90% suppression at the highest concentration (72 h). Fig 8 shows the dose-and-time dependent cytotoxic effects of POP on EAC cells.

## Conclusion

The ideal polysaccharide extraction states of water/crude material proportion, ultrasound time, and ultrasound control were determined utilizing the RSM of BBD in view of single factor tests. The RSM of BBD could effectively improve the extraction states of POP, and the regression model was pertinent for removing POP. The antioxidant activity of DPPH and ABTS radicals’ in vitro measures demonstrate that POP has cancer prevention agent properties that increase with the increasing convergence of the polysaccharide arrangement. Hence, these outcomes suggest that polysaccharide from *Pleurotus ostreatus (Jacq*.*)* P. Kumm ought to be further investigated as a novel and potential natural antioxidant, which may prove a useful therapeutic drug. Additionally the purification and structure identification are improve the overall understanding of polysaccharide.

## Supporting information

S1 Dataset Impact of three variables on the POP extraction from Pleurotus ostreatus. (XLSX)

S2 Dataset Antioxidant activities of the POP. (XLSX)
